# Drilling Rate of Penetration Prediction Based on CBT-LSTM Neural Network

**DOI:** 10.3390/s24216966

**Published:** 2024-10-30

**Authors:** Kai Bai, Siyi Jin, Zhaoshuo Zhang, Shengsheng Dai

**Affiliations:** 1 Hubei Key Laboratory of Drilling and Production Engineering for Oil and Gas, Yangtze University, Wuhan 430100, China; 2 School of Computer Science, Yangtze University, Jingzhou 434023, China; 2022710655@yangtzeu.edu.cn (S.J.); 2022710626@yangtzeu.edu.cn (Z.Z.); 2022710651@yangtzeu.edu.cn (S.D.)

**Keywords:** ROP prediction, 2D-CNN, BiLSTM, temporal pattern attention mechanism, deep learning

## Abstract

Due to the uncertainty of the subsurface environment and the complexity of parameters, particularly in feature extraction from input data and when seeking to understand bidirectional temporal information, the evaluation and prediction of the rate of penetration (ROP) in real-time drilling operations has remained a long-standing challenge. To address these issues, this study proposes an improved LSTM neural network model for ROP prediction (CBT-LSTM). This model integrates the capability of a two-dimensional convolutional neural network (2D-CNN) for multi-feature extraction, the advantages of bidirectional long short-term memory networks (BiLSTM) for processing bidirectional temporal information, and the dynamic weight adjustment of the time pattern attention mechanism (TPA) for extracting crucial information in BiLSTM, effectively capturing key features in temporal data. Initially, data are denoised using the Savitzky–Golay filter, and five correlation coefficient methods are employed to select input features, with principal component analysis (PCA) used to reduce model complexity. Subsequently, a sliding window approach transforms the time series into a two-dimensional structure to capture dynamic changes, constructing the model input. Finally, the ROP prediction model is established, and search methods are utilized to identify the optimal hyperparameter combinations. Compared with other neural networks, CBT-LSTM demonstrates superior performance metrics, with MAE, MAPE, RMSE, and *R*^2^ values of 0.0295, 0.0357, 9.3101%, and 0.9769, respectively, indicating the highest predictive capability. To validate the model’s robustness, noise was introduced into the training data, and results show stable performance. Furthermore, the model’s predictive results for other wells achieved R^2^ values of 0.95, confirming its strong generalization ability. This method provides a new solution for ROP prediction in real-time drilling operations, assisting drilling engineers in better planning their operations and reducing drilling cycles.

## 1. Introduction

In the oil industry, drilling is the process of creating long and narrow boreholes using tools such as drill bits, drill strings, and drilling fluids, and it serves as a critical step in the exploration and development of petroleum resources [1]. Optimizing the rate of penetration (ROP) contributes to reducing drilling cycles and costs; thus, establishing a high-accuracy ROP prediction model is of significant importance [2]. ROP is influenced by various factors, including controllable factors (such as drill bit type, weight on bit, and rotation speed) and uncontrollable factors (such as formation lithology and formation pressure) [3]. High-precision ROP prediction models help minimize downtime and drilling costs. Therefore, establishing an effective rate of penetration (ROP) prediction model can not only help describe changes in ROP but also significantly improve drilling efficiency.

Early prediction methods primarily relied on physical laws and empirical rules, such as the models proposed by Galle and Woods (1963), Bingham (1965), and Bourgoyne and Young (1974) [4], which were established through mathematical formulas or physical experiments. However, due to the complexity of drilling environments, these models struggled to accurately capture the nonlinear relationships among multiple variables, resulting in suboptimal predictive performance. With advancements in artificial intelligence technology, deep learning-based models for rate of penetration (ROP) prediction have gradually emerged. These models, particularly deep learning architectures, have demonstrated strong generalization performance, prompting many researchers to seek improvements for their application in ROP prediction [5]. Ashrafi et al. [6] combined particle swarm optimization (PSO) and genetic algorithms (GA) with artificial neural networks (ANNs), finding that this hybrid ANN significantly outperformed traditional backpropagation-trained ANNs in accuracy. Similarly, AI-AbdulJabbar et al. [7] integrated an adaptive differential evolution optimization algorithm with ANNs for ROP prediction in carbonate reservoirs. AI-based models can effectively fit the complex nonlinear relationships present in drilling data, markedly surpassing traditional physical models. However, ANN-based ROP prediction models typically handle static data, neglecting the temporal dynamics of drilling operations, which impacts their effectiveness in practical applications.

As understanding of drilling time series data has deepened, researchers have begun to incorporate time series models for ROP prediction. Recurrent neural networks (RNNs) have been widely used in ROP prediction due to their ability to capture historical information [8]. Encinas et al. [9] combined RNNs with multilayer perceptions, significantly improving ROP prediction accuracy by leveraging the sequential nature of drilling operations, achieving better results compared with traditional machine learning models like random forests. Etesami et al. [10] validated the potential of RNNs within a drilling training framework, although their prediction accuracy reached only 0.90, indicating room for further optimization in ROP prediction tasks. These studies highlight that, despite their effectiveness in certain contexts, RNNs face significant limitations in capturing long-term dependencies due to issues like vanishing and exploding gradients when processing long sequences. This has prompted researchers to explore more advanced models, such as long short-term memory networks (LSTMs), to enhance predictive performance.

Compared with ANNs and RNNs, LSTMs are particularly adept at handling long-term dependency information, owing to their memory cell structure, which enables outstanding performance in ROP prediction [11]. Safarov et al. [12] conducted a thorough comparison of traditional machine learning and deep learning methods (such as RNNs and LSTMs) in ROP prediction; however, their study did not delve deeply into data partitioning, a critical factor for achieving accurate ROP predictions. Liu et al. [13] designed a model that connects LSTM and RNN for ROP prediction, but their research focused solely on deep wells, raising questions about the model’s generalization capability. Zhang et al. [14] proposed a model combining generative adversarial networks (GANs) with LSTM to predict ROP in continuous coiled tubing operations. However, LSTMs can only utilize input information from prior time points during prediction and cannot integrate outputs from the entire time series. This limitation of unidirectional analysis prevents traditional LSTM models from adequately addressing the complex nonlinear relationships and temporal dynamics among drilling parameters. While LSTMs offer improvements over other models, further exploration and optimization are still necessary in certain aspects.

Bidirectional long short-term memory networks (BiLSTMs), as an extension of unidirectional LSTM networks, can capture bidirectional information within time series, thereby enhancing the accuracy of temporal tasks [15]. For instance, Kocoglu et al. [16] found that BiLSTM outperformed traditional and other deep learning methods in predicting production from multiple wells in the Marcellus formation. Liang et al. [17] proposed a hybrid model combining BiLSTM and random forests (RF) for shale gas production forecasting, successfully addressing complex nonlinear and non-stationary characteristics. Given the extended time span of drilling data, the use of neural networks for prediction may result in the neglect of bidirectional information due to long-term dependencies, ultimately affecting prediction accuracy. BiLSTM not only effectively captures contextual information from logging curves but also enhances the model’s sensitivity to changes in nonlinear features, thereby improving data utilization and predictive performance.

The introduction of bidirectional computation in BiLSTM effectively enhances the accuracy of temporal tasks; however, it also increases model complexity and training costs. To address the computational burden associated with BiLSTM, attention mechanisms have been incorporated into ROP prediction. The attention mechanism dynamically optimizes the weight allocation of input features, highlighting key components and thereby improving the model’s efficiency and accuracy. Cheng et al. [18] combined the attention mechanism with LSTM, resulting in a significant improvement in the model’s predictive performance. Similarly, Song et al. [19] proposed an attention-based BiLSTM model for forecasting wind and wave energy, demonstrating that the attention mechanism markedly enhanced the model’s performance. Although traditional attention mechanisms excel in optimizing model performance, they still face challenges, such as high computational complexity and attention diffusion when dealing with long-term dependencies [20]. To address these issues, the temporal pattern attention (TPA) mechanism was introduced [21]. By dynamically allocating weights across the time series, TPA captures key temporal information, further enhancing model prediction accuracy. In ROP prediction tasks, TPA utilizes a time-series-based attention weight matrix to help BiLSTM more effectively capture important features within the contextual information, significantly improving both prediction accuracy and efficiency.

The combination of convolutional neural networks (CNN) and long short-term memory (LSTM) networks offers significant advantages in feature extraction and has been widely applied to time series prediction tasks [22]. CNN effectively extracts features from time series data through progressive convolution and pooling operations, while LSTM selectively updates and outputs information from the memory cells using its gating mechanism. This hybrid structure enhances the model’s ability to capture data features, thereby improving the effectiveness of time series learning [23]. In multivariate time series data, there is often local correlation between different variables. Compared with 1D-CNN, which can only extract features along a single dimension, two-dimensional convolutional neural networks (2D-CNN) can perform convolution operations simultaneously across both the time steps and variable dimensions. This advantage allows 2D-CNN to more effectively capture relationships between multiple variables, making it particularly well-suited for complex multivariate time series analysis. For instance, Jonkers et al. [24] combined 2D-CNN with conformal quantile regression (CQR) for regional wind power forecasting and found that the model, when handling high-dimensional input data, exhibited fewer parameters and lower computational complexity compared with transformer models. Additionally, the 2D-CNN model embedded with Laplacian attention proposed by Tuyen et al. [25] demonstrated the potential to analyze input sequence features from multiple perspectives. Thus, 2D-CNN not only performs convolutions across features and time steps but also effectively handles the complex multidimensional characteristics present in drilling tasks. This capability significantly enhances the model’s ability to capture nonlinear relationships, thereby improving overall predictive performance. Compared with 1D-CNN, the feature extraction advantages of 2D-CNN affirm its suitability for complex time series forecasting tasks.

Despite significant progress in ROP prediction using deep learning, existing studies still face the following challenges:

(1) Excessive redundancy exists among input data related to the rate of penetration (ROP), with varying contributions to ROP prediction deriving from different features, which can easily lead to model overfitting.
(2) The complex nonlinear relationships between input variables have not been fully explored, and the effectiveness of feature extraction requires improvement.
(3) As a time-series data problem, traditional methods have limitations in extracting long-term dependencies, which negatively affects prediction accuracy. These issues collectively reduce the predictive capability of the models.

To address the aforementioned challenges, leveraging the complementary strengths of 2D-CNN and BiLSTM networks is crucial. BiLSTM excels at capturing temporal features from sequential data but has limited capacity in extracting nonlinear relationships between input features, especially when dealing with complex input parameters, which can lead to reduced prediction accuracy. In contrast, 2D-CNN offers strong nonlinear feature extraction capabilities, yet its local sensitivity and inductive bias limit its ability to fully utilize global information in time-series data. Therefore, combining these two models can facilitate the efficient extraction and analysis of both global and local features within the data, leading to improved predictive performance.

In summary, fully exploring feature correlations and bidirectional temporal information within drilling data is critical to improving the accuracy of ROP prediction. To address these challenges and provide more precise predictions, this study proposes an improved hybrid prediction model—CBT-LSTM. The model is based on experimental research conducted using data from four wells in a Chinese oilfield. Results indicate that the CBT-LSTM model outperforms other models in most cases, demonstrating superior predictive accuracy.

The main contributions of this paper include:

(1) Proposing a hybrid model (CBT-LSTM) that integrates 2D-CNN, BiLSTM, and the temporal pattern attention (TPA) mechanism. In this model, 2D-CNN is used to extract nonlinear feature relationships from the input data, BiLSTM enhances the global understanding of time-series data, and TPA further improves BiLSTM’s focus on key temporal features by reducing redundant information, thereby enhancing prediction accuracy.
(2) Conducting comparative experiments with traditional neural network methods and testing on wells of different depths. The CBT-LSTM model demonstrated superior performance in metrics such as mean absolute error (MAE), mean absolute percentage error (MAPE), root mean square error (RMSE), and *R*^2^, indicating its significant feasibility and strong generalization capability.
(3) Validating the model’s robustness by introducing 10%, 20%, and 30% noise and missing values into the training set. The results show that the model maintains strong predictive performance even in high-noise environments, supporting its practical applicability in real-world scenarios.

The remainder of this paper is structured as follows. Section 2 provides a detailed description of the research methodology and the architecture of the CBT-LSTM model. Section 3 outlines the general steps involved in ROP prediction, including data preprocessing, the construction of the sliding window, and the model evaluation metrics. Section 4 presents the selection of hyperparameters for the CBT-LSTM model, a discussion and analysis of the experimental results, and highlights the limitations and directions for future research. Finally, Section 5 summarizes the paper and presents the conclusions.

## 2. Theory

### 2.1. Two-Dimensional Convolutional Neural Network (2D-CNN)

CNNs are a type of deep feedforward network commonly used for processing time series, images, and audio data [26,27,28,29]. By reshaping the input dataset into a 2D format, cross-variable features can be considered during the convolution operations. Traditional time series prediction methods primarily rely on the autocorrelation of the sequence itself, while 2D-CNNs integrate the time dimension into the input, allowing for more comprehensive extraction of feature information from the time series.

**Figure 1:**
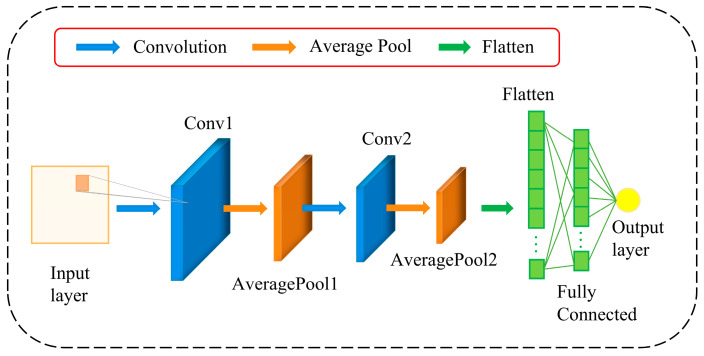
A general structure of 2D-CNN.

Figure 1 shows the architecture of the 2D-CNN. A CNN consists of two main parts: the feature extraction part (convolutional layers, pooling layers) and the learning part (fully connected layers). The convolutional layer and activation function are the core components of a convolutional neural network. The convolutional layer extracts local features from the input data through convolution operations. By applying convolutional kernels (filters) to each position in the input data, a feature map is generated through a dot product operation. The activation function introduces nonlinearity, thereby enhancing the model’s expressive capability. Because the convolution operation itself is linear, the activation function helps the model learn more complex mapping relationships. ReLU is one of the most widely used activation functions; it transforms negative input values into 0 while keeping positive inputs unchanged. Its advantages include simplicity of computation, mitigation of the vanishing gradient problem, and acceleration of model convergence.

Convolution operations are the core of CNNs, where kernels slide over the input data, extracting local features by performing convolution operations on each window. By using multiple kernels, the network can learn different features, thereby capturing the complex structure of the input data. The convolutional layer can be represented as follows:

(1) $$c_{i}=f\left(w_{i}\cdot x_{i}+b_{i}\right)$$

In the equation, $x_{i}$ represents the input of the *i*-th layer, $c_{i}$ is the output of the *i*-th layer, $w_{i}$ is the weight matrix, $b_{i}$ is the bias vector, and $f\left(\cdot \right)$ denotes the activation function.

The pooling layer is used to reduce the output dimensions, decrease the number of parameters, and retain critical information. Here, average pooling is chosen to select the mean value from each region, as expressed in Equation (2):

(2) $$avg_{pool\left(x_{i}\right)}=\frac{1}{n}\sum_{k=1}^{n}x_{i-1}^{k}$$

where $x_{i-1}^{k}$ represents the activation value of the *k*-th neuron in one channel of the (*i* − 1)-th layer and *n* is the number of elements within the pooling window.

Finally, the fully connected layer is responsible for mapping the features obtained from convolution and pooling to the final output.

### 2.2. Bidirectional Long Short-Term Memory Network (BiLSTM)

**Figure 2:**
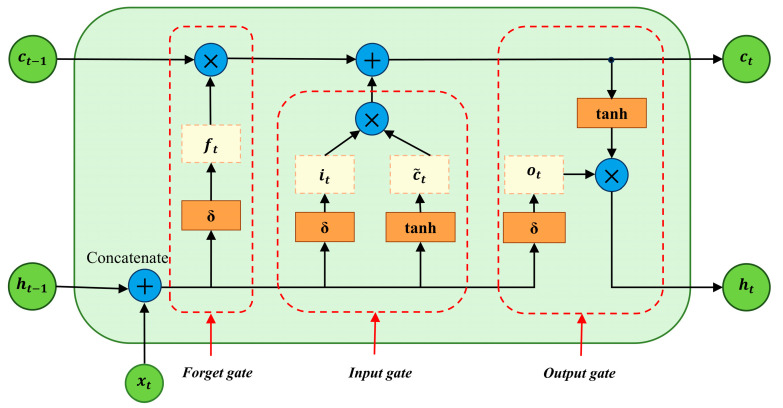
Logical structure diagram of the LSTM neural network.

LSTM is an optimized algorithm for RNN, effectively addressing the issues of memory retention and gradient vanishing in traditional RNNs. LSTM manages the retention and forgetting of information through the introduction of controllable gate mechanisms (forget gate, input gate, and output gate). The basic unit of LSTM is shown in Figure 2, where δ represents the sigmoid activation function, and tanh represents the tanh activation function. The LSTM algorithm implementation involves three steps.

First, the forget gate $f_{t}$ controls how much of the past memory $c_{t-1}$ is retained. The input consists of the previous output and the current input $\left[h_{t-1},x_{t}\right]$, with the output range being (0, 1), as shown below:

(3) $$f_{t}=\delta \left(W_{f}\times \left[h_{t-1},x_{t}\right]+b_{f}\right)$$

Second, the input gate $\;i_{t}$ controls how much new data are adopted at the current time step, creating new candidate memory ${\tilde{c}}_{t}$. The input gate uses the sigmoid function to control the amount of input, as shown in Equation (4). A new candidate value is created using the tanh function and added to the candidate state, as shown in Equation (5).

(4) $$i_{t}=\delta \left(W_{i}\times \left[h_{t-1},x_{t}\right]+b_{i}\right)$$

(5) $${\tilde{c}}_{t}=\tanh\left(W_{c}\times \left[h_{t-1},x_{t}\right]+b_{c}\right)$$

After processing through the forget gate and the input gate, the memory cell is updated to $c_{t}$, as shown below:

(6) $$c_{t}=c_{t-1}⨂f_{t}+i_{t}⨂{\tilde{c}}_{t}$$

Third, the output gate $o_{t}$ controls how much of the current memory $c_{t}$ is output to the external state $h_{t}$.

(7) $$o_{t}=\delta \left(W_{0}\times \left[h_{t-1},x_{t}\right]+b_{0}\right)$$

(8) $$h_{t}=\tanh\left(c_{t}\right)⊗o_{t}$$

**Figure 3:**
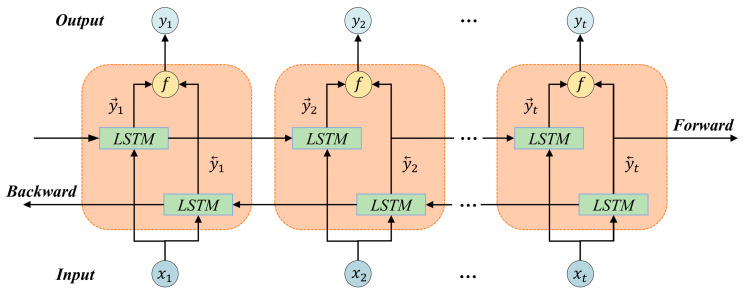
BiLSTM network architecture.

Traditional LSTM networks can only utilize preceding contextual information [30]. To access distant information, Graves and Schmidhuber proposed the bidirectional LSTM (BiLSTM) to better capture contextual dependencies in both direction [31]. The bidirectional architecture simultaneously extracts contextual information from both directions through forward and backward hidden layers, enhancing feature extraction efficiency and performance. The structure of BiLSTM is shown in Figure 3.

In Figure 3, ${\vec{h}}_{t}$ and ${\overset{\leftarrow }{h}}_{t}$ are the outputs of the forward and backward hidden layers, respectively. The output and hidden sequences of the forward layer are iteratively calculated from step 1 to step t, while those of the backward layer are iteratively calculated from step t to step 1. ${\vec{h}}_{t}$ and ${\overset{\leftarrow }{h}}_{t}$ are obtained through standard LSTM calculations. The BiLSTM layer produces an output vector Y, where each element is calculated according to Equation (11), as follows:

(9) $${\vec{h}}_{t}=LSTM\left(x_{t},{\vec{h}}_{t-1}\right)$$

(10) $${\overset{\leftarrow }{h}}_{t}=LSTM\left(x_{t},{\overset{\leftarrow }{h}}_{t-1}\right)$$

(11) $$y_{t}=f\left({\vec{h}}_{t},{\overset{\leftarrow }{h}}_{t}\right)$$

where the function f couples the sequences ${\vec{h}}_{t}$ and ${\overset{\leftarrow }{h}}_{t}$. The function f can be a sum function, a multiplication function, a concatenation function, or an averaging function. The final output of the BiLSTM layer can be represented as the vector Y = [$y_{1}$,$y_{2}$,…,$y_{t}$].

### 2.3. Temporal Pattern Attention (TPA)

**Figure 4:**
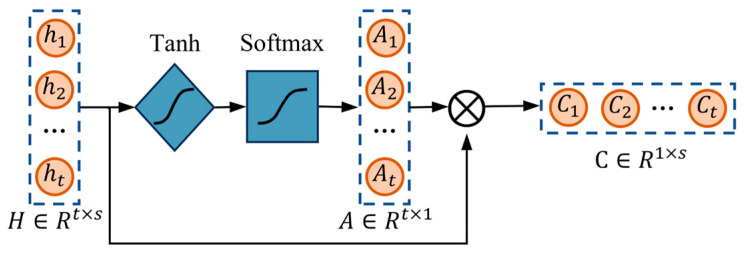
TPA structure.

Unlike traditional attention mechanisms, the temporal pattern attention (TPA) mechanism focuses more on the influence of historical inputs on the current time step [32]. The temporal attention module is adaptive and capable of extracting temporal dependencies between inputs. In the channel dimension, meaningful historical information is enhanced, and the impact of this information is further optimized through learned weight assignments. This approach is particularly suitable for analyzing multivariate time series data and helps improve the model’s predictive performance and accuracy. The structure is shown in Figure 4.

First, the hidden state output sequence H is obtained from the BiLSTM units, where t is the number of time steps and s is the feature dimension.

(12) $$H={\left[h_{1},h_{2},\ldots ,h_{t}\right]}_{t\times s}$$

When constructing the attention mechanism, it is necessary to initialize two trainable parameters: the weight matrix W and the bias vector b. W is used for the linear transformation of input features, and b adjusts the results of the linear transformation. These parameters will be continuously updated during training. For the input sequence H, the attention scores S are calculated for each time step, reflecting the importance of each time step.

(13) $$S=\tanh\left(H⊙W+b\right)$$

Next, the attention weights A are computed using the softmax function to perform normalization over the time steps (axis = 1). This ensures that the attention weights assigned to different time steps reasonably reflect their importance. The calculation formula is shown in Equation (14).

(14) $$A=softmax\left(S,\;axis=1\right)$$

Finally, the attention weights A are used to weight the input sequence H, resulting in the context vector C, as follows:

(15) C=A⊙H

### 2.4. CBT-LSTM Model Structure Design

**Figure 5:**
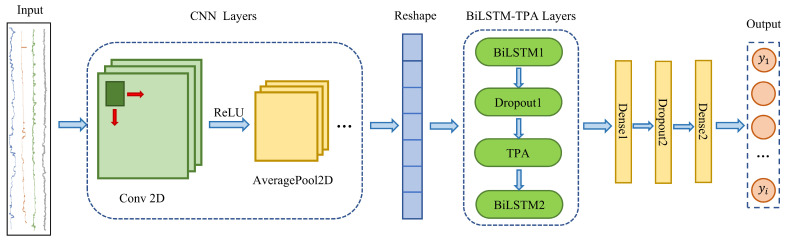
The structure of the CBT-LSTM model.

This paper constructs a CBT-LSTM model to predict ROP, and the model structure is shown in Figure 5. First, the preprocessed data are input into the network with the shape (number of samples, sliding window width, number of features). The network utilizes 2D-CNN for feature extraction, including convolution and pooling operations, with the ReLU function employed as the activation function. After each convolution operation, an average pooling layer follows. The network undergoes three layers of convolution and pooling operations in total. Next, the three-dimensional data are reshaped into two-dimensional data and fed into the BiLSTM network. The BiLSTM network, designed for handling long sequence data, includes two hidden layers in this model, each containing 64 units and using the tanh activation function. To enhance the model’s temporal modeling capability, a TPA module is inserted between the two BiLSTM layers. This module assigns importance to different features in the input sequence. It automatically learns and evaluates the significance of various features, and assigns weights based on their importance. To prevent overfitting, two dropout layers are added to the model, randomly dropping some neurons. Finally, the output prediction is obtained through two fully connected layers that use ReLU and linear activation functions, respectively.

## 3. Methodology

**Figure 6:**
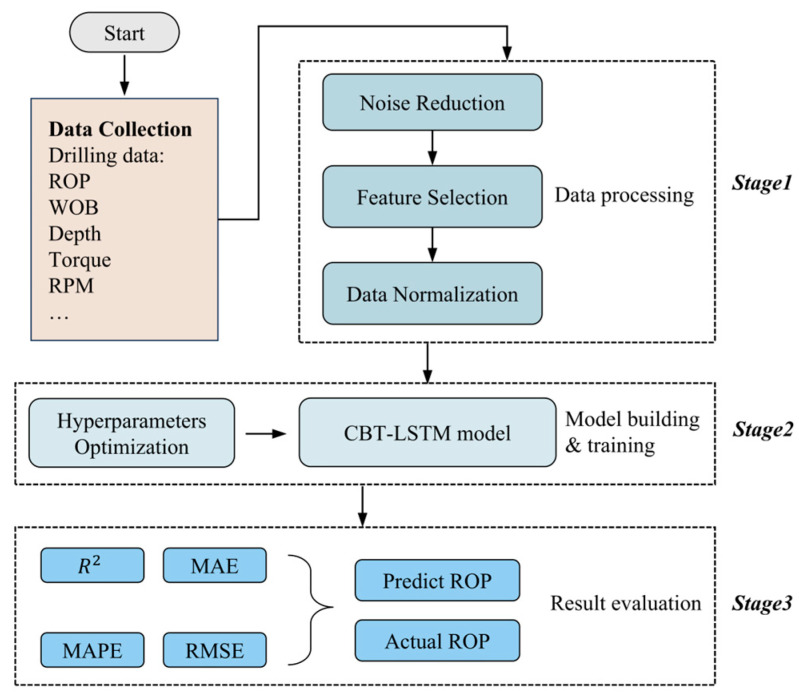
Workflow chart of ROP forecasting.

The workflow for ROP prediction is mainly divided into three primary steps. The first step is data preprocessing and feature selection. The second step involves model building and training. The final step is model evaluation, as shown in Figure 6.

### 3.1. Data Collection and Description

The dataset for this study is sourced from four vertical wells in an oilfield located in southwestern China, all of which utilize the same bottom hole assembly (BHA) for drilling. To achieve real-time prediction of the ROP, various real-time sensors are relied upon to gather key drilling parameters. Pressure sensors are employed to monitor changes in mud pressure, bottom hole pressure, and casing pressure, ensuring pressure balance within the well and preventing dangerous situations such as blowouts. Drilling speed sensors reflect real-time drilling progress by measuring the rate at which the drill bit penetrates the formation. Logging while drilling (LWD) sensors are used to measure the physical properties of the formation, such as gamma rays. Additionally, mud flow sensors monitor the flow rate of the drilling fluid in real time, ensuring the cleanliness of the borehole and preventing wall collapse or stuck drill bits. Through the coordinated operation of these sensors, the safety and operational efficiency of the drilling process can be effectively enhanced.

The dataset in this study strictly follows depth-based sequential data, with each row measured in meters. The depth range and data entries of the wells are shown in Table 1. The depths of the wells vary to ensure dataset diversity. Well A has the most extensive data and a relatively wide depth range, which helps the model better learn and understand geological features. The other wells, with different depth ranges, are used as validation sets for assessing the model’s generalization performance under varying geological conditions. Table 2 provides detailed information on all of the parameters present in Well A.

### 3.2. Data Preprocessing

#### 3.2.1. Data Cleaning

Outliers and noisy samples in the dataset can negatively impact model performance. Outliers refer to samples with unreasonable or illogical values. For example, in the dataset used in this study, samples where the weight on bit (WOB) is zero while the ROP is greater than zero; samples where the mud flow rate, mud density, or average surface torque are below zero; and samples where the ROP exceeds 100 m/h or the average standpipe pressure exceeds 25,000 kPa, are considered outliers that do not align with logical or expected values. These outliers must be identified and manually removed from the dataset to ensure the accuracy and reliability of the model.

**Figure 7:**
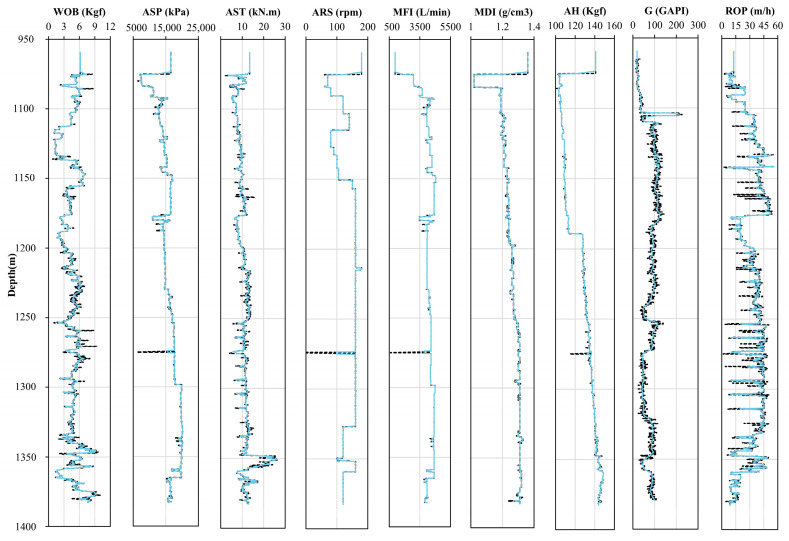
Graph comparing measured data (black lines) with denoised data (blue lines) for well A.

To reduce noise in the data, various methods can be employed, such as low-pass filters, moving averages, wavelet transforms, and the Savitzky–Golay (SG) smoothing filter [33]. In this study, the Savitzky–Golay (SG) smoothing filter was used for denoising [34]. The SG technique is a commonly used smoothing method in geosciences, widely applied for noise reduction in petroleum logging data [6,35]. The SG filter effectively removes noise while preserving the underlying data features and trends, avoiding the potential information loss associated with simpler averaging methods. This filter smooths the data by fitting an n-th order polynomial to the data within a specified window, reducing noise. The choice of polynomial order and window size is crucial to the filter’s effectiveness. Therefore, the SG filter was employed in this study as a denoising tool to enhance the model’s predictive accuracy. After evaluation, the optimal polynomial order was determined to be 3, and the optimal window size was 39. Figure 7 compares the raw data with the denoised data, showing that the denoised data retains the trend of the original records while minimizing noise impact.

#### 3.2.2. Feature Extraction

Traditional methods for predicting ROP often lack systematic parameter selection, and the impact of different parameters on ROP is inconsistent. The interrelationships among data can affect both the training speed and effectiveness of the model; therefore, reasonable selection of input features is necessary before training. Over the past few decades, significant factors influencing ROP have been identified through theoretical analysis, laboratory experiments, and field observations, leading to the development of various physics-based ROP models. Among these factors, weight on bit and rotary speed are the most critical influences and must be considered as feature variables [36]. Based on the understanding of these key influencing factors, conducting a correlation analysis will help quantify the relationship between each feature and ROP.

**Figure 8:**
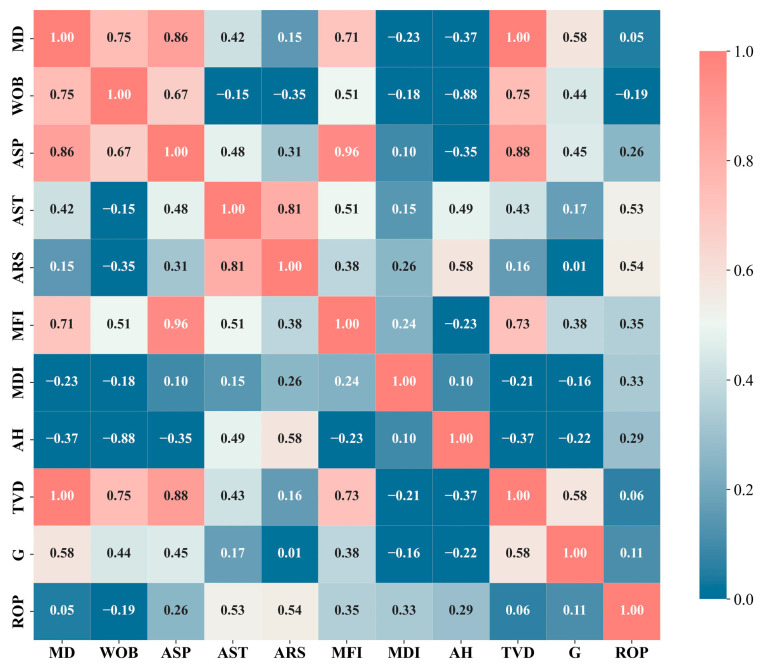
Pearson correlation coefficient heat map.

First, the Pearson correlation coefficient [37] was used to assess the linear correlations between various features and the rate of penetration (ROP). The range of the Pearson correlation coefficient *r* is [−1, 1], where a positive *r* indicates a positive correlation and a negative *r* indicates a negative correlation. According to the results shown in Figure 8, parameters such as AST, ARS, MFI, and MDI exhibit strong correlations with ROP, and are thus selected as input features. However, though MD, WOB, ASP, AH, TVD, and G have lower correlation coefficients, this does not imply that their influence on ROP is insignificant. They may affect ROP indirectly or through interactions with other factors, making it necessary to further analyze their nonlinear relationships.

To evaluate the nonlinear relationships between these variables and the rate of penetration (ROP), the Spearman correlation coefficient [38], Kendall correlation coefficient [39], distance correlation coefficient [40], and maximum information coefficient [41] were utilized. The Spearman correlation coefficient (Sc) and Kendall correlation coefficient (Kc) measure the monotonic nonlinear relationships between two variables, both ranging from [−1, 1], where 1 indicates perfect monotonic positive correlation, −1 indicates perfect monotonic negative correlation, and 0 indicates no monotonic relationship. The distance correlation coefficient (Dc) and maximum information coefficient (Mc) capture any dependency relationship between variables, including both linear and nonlinear relationships, with a range of [0, 1], where 0 indicates complete independence and 1 indicates complete correlation. Table 3 presents the nonlinear correlation coefficients between MD, WOB, ASP, AH, TVD, G, and ROP. Considering these results collectively, these six variables can also be included as input features for the model. Based on the above analysis, this study ultimately selects AST, ARS, MFI, MDI, MD, WOB, ASP, AH, TVD, and G as the feature variables for the model.

According to the analysis in Figure 8, there is a high correlation between MD and WOB, as well as between ASP. Similarly, there is a strong correlation between AST and ARS. These highly correlated features may negatively impact the model’s generalization ability and potentially lead to overfitting. Therefore, it is necessary to evaluate and select which features to retain. To reduce redundant features in the model while preserving essential information, principal component analysis (PCA) was employed for dimensionality reduction [42]. PCA simplifies the model while improving its stability and generalization ability by transforming multiple correlated variables into a smaller set of uncorrelated principal components, retaining the most critical information from the data. The core idea of PCA is to identify new orthogonal directions (called principal components, PCs) such that the projected data along these directions have the largest variance, thereby preserving the maximum amount of information. Specifically, the principal components are linear combinations of the original variables and are mutually independent. By calculating the eigenvalues and eigenvectors of the covariance matrix, PCA selects the eigenvectors corresponding to the largest eigenvalues as the principal components, effectively retaining the primary structure and features of the data.

**Figure 9:**
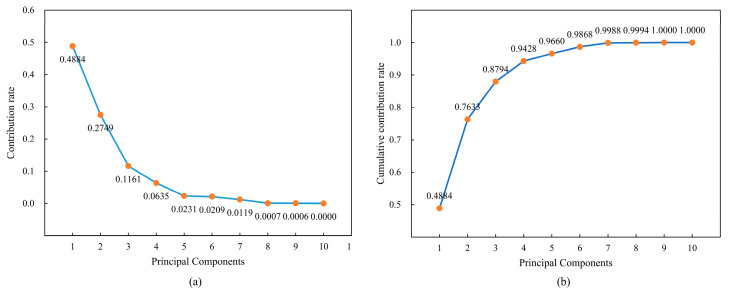
Contribution rates and cumulative contribution rates of the principal components in Well A. (**a**) Variation of different PCs across 10 features. (**b**) Cumulative contribution rates of the principal components for the 10 features.

As shown in Figure 9, the first six principal components (PC1 to PC6) account for 98.68% of the total variance in the dataset. In contrast, the remaining four PCs account for only 1.32%. Therefore, the original 10-dimensional dataset (with 10 variables) can be reduced to a 6-dimensional dataset without significant information loss.

#### 3.2.3. Normalization

Data normalization is crucial for model training. Drilling log data have a wide range of values that need to be scaled to the same range to speed up the learning process. Additionally, normalization can accelerate the convergence process when using gradient-based optimization algorithms (such as Adam), helping the model find the optimal solution more quickly [43]. Therefore, the min–max scaling method is used to scale the input sequence to a range of 0–1, where $x_{i}$ is the original data and $y_{i}$ is the normalized data, as shown in the following formula:

(16) $$y_{i}=\frac{\left(x_{i}-\bar{x}\right)}{\left(x_{max}-x_{min}\right)}$$

### 3.3. Sliding Window Technique for Drilling Data Processing

**Figure 10:**
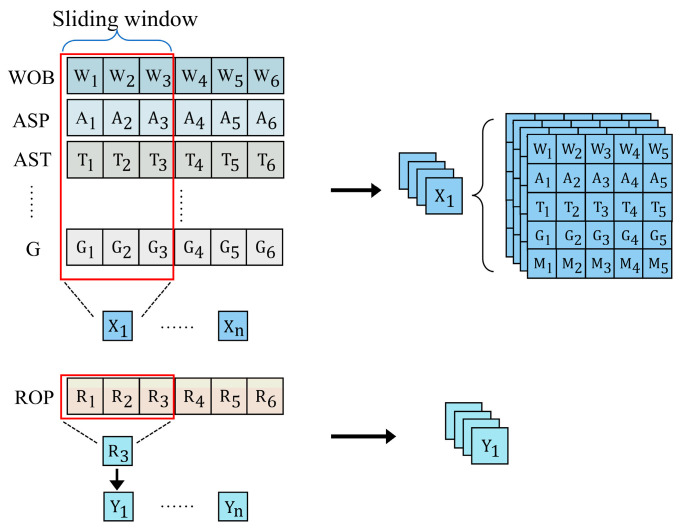
Sliding window strategy for data partitioning and concatenation.

In this study, the sliding window technique is employed to process drilling data, preserving the temporal characteristics of the data and fitting the input layer of the 2D convolutional neural network (CNN). The size of the sliding window determines the number of data points at each time step, enabling the model to capture the relationships between preceding and subsequent data points in the sequence, thus improving the accuracy of time series prediction. As illustrated in Figure 10, the sliding window moves over both feature variables and target variables.

The size of the sliding window significantly impacts the processed dataset. As the window size increases, the number of input features rises, which affects the feature extraction performance of the CNN model. Consequently, this impacts the overall prediction accuracy and computation time [44]. Therefore, the window width is set to 20, with a stride of 1. The dataset partitioned in this manner is then subjected to ten-fold cross-validation to maximize data utilization, reduce overfitting, and ensure the stability and generalization ability of the model.

### 3.4. Evaluation Metrics

The CBT-LSTM model is compared with other predictive models using the same dataset. The effectiveness of the proposed method is evaluated using metrics such as the coefficient of determination (*R*^2^), mean absolute error (MAE), mean absolute percentage error (MAPE), and root mean square error (RMSE). An *R*^2^ value closer to 1 indicates a better fit of the model to the data, while smaller values of MAE, MAPE, and RMSE indicate better model performance. The formulas for these evaluation metrics are as follows:

(17) $$R^{2}=1-\frac{\sum_{i=1}^{n}{\left(y_{i}-{\hat{y}}_{i}\right)}^{2}}{\sum_{i=1}^{n}{\left(y_{i}-\bar{y}\right)}^{2}}$$

(18) $$MAE=\frac{1}{n}\sum_{i=1}^{n}\left|y_{i}-{\hat{y}}_{i}\right|$$

(19) $$MAPE=100\%\times \frac{1}{n}\sum_{i=1}^{n}\left|\frac{y_{i}-{\hat{y}}_{i}}{y_{i}}\right|$$

(20) $$RMSE=\sqrt{\frac{1}{n}\sum_{i=1}^{n}{\left(y_{i}-{\hat{y}}_{i}\right)}^{2}}$$

where n represents the total number of samples, $y_{i}$ denotes the actual values, ${\hat{y}}_{i}$ denotes the predicted values, and $\bar{y}$ represents the mean of the actual values across the n samples.

## 4. Experiments, Results, and Discussion

### 4.1. The Impact of Different Convolution Kernel Combinations on 2D-CNN Modeling

When constructing deep learning models, the configuration of neuron parameters is crucial, as different settings can significantly affect the overall performance of the model. In 2D-CNN, multiple convolution kernels are used to learn various features, effectively addressing the multivariate correlation issues in drilling data. Therefore, the size and number of convolution kernels play a key role in the performance of the CNN model. To determine the optimal convolution kernel configuration, experiments were conducted to compare the model’s performance under different settings. Specifically, the number of layers in the 2D-CNN was fixed at three, with traditional 3 × 3 convolution kernels, as well as 2 × 1 and 3 × 1 kernels, being selected to reduce computational cost, as per [45]. To simplify the network design, the stride was set to the default value of 1, and zero padding was applied. Additionally, the number of convolution kernels was kept consistent across the three convolutional layers. As shown in Table 4, when the convolution kernel size was 2 × 1 and the number of kernels was 64, the model achieved the highest *R*^2^ value of 0.9624, indicating the best predictive performance.

### 4.2. Hyperparameter Optimization

All experiments were conducted on a Windows system with an Intel i5-12400 CPU (Manufacture: Intel, Santa Clara, CA, USA), 32 GB of RAM (Manufacture: KINGBANK, Shenzhen, China), and an NVIDIA GeForce RTX 3060 12 GB GPU (Manufacture: NVIDA, Santa Clara, CA, USA). The development was performed using Python 3.9 and TensorFlow 2.10.

To find the optimal combination of model hyperparameters, grid search, random search, and particle swarm optimization (PSO) were used for hyperparameter tuning. Grid search systematically evaluates each combination of hyperparameters within a predefined range by exhaustively searching the entire space. This approach ensures that the best configuration is found, but can be computationally expensive, especially for large search spaces. Random search selects hyperparameter combinations randomly within the defined parameter space. Compared with grid search, random search is more efficient at exploring large-scale search spaces, as it can potentially locate near-optimal configurations with less computational effort. Particle swarm optimization (PSO) is an optimization algorithm that simulates the foraging behavior of bird flocks. It leverages swarm intelligence to dynamically adjust particle positions in the search space, allowing for efficient exploration and identification of the optimal solution. PSO is particularly advantageous in complex and multi-dimensional search spaces, offering a balance between exploration and exploitation.

In this study, the selected hyperparameters for optimization are batch size, learning rate, dropout rate of the first dropout layer, and dropout rate of the second dropout layer. The approximate range of these hyperparameters was determined based on relevant literature, with the specific search ranges provided in Table 5. The number of iterations was set to 100 to ensure sufficient optimization space. When applying PSO, the particle count was set to 40, with an inertia weight of 0.7, an individual learning factor of 0.5, a social learning factor of 2.5, and a maximum of 100 iterations, as per [2]. These settings aim to strike a balance between convergence speed and exploration of the search space, ensuring the model reaches optimal hyperparameter configurations effectively.

The hyperparameter tuning results obtained from the three search methods are shown in Table 6. From the perspective of model accuracy, comparing *R*^2^ and MAE values, the PSO method performed the best, while random search and grid search yielded similar results. This indicates that PSO more effectively enhances the neural network model’s accuracy and is recommended over the other two methods. In the hyperparameter combination derived from PSO, the batch size was 64, the learning rate was 0.005, dropout1 was 0.2, and dropout2 was 0.2. Under this hyperparameter configuration, the model achieved the highest *R*^2^ value of 0.9684, indicating optimal predictive performance. Therefore, in all subsequent experiments, the model’s hyperparameters were fixed to these optimal values.

### 4.3. Cross-Validation Method and Performance Analysis

During model development, cross-validation is commonly used as it allows for more efficient utilization of data samples, increasing the frequency of both training and validation. This generates more predictive results, helping to identify the optimal model parameters and reducing the risk of overfitting. Among these methods, K-fold cross-validation divides the dataset *D* into *K* approximately equal and mutually exclusive subsets, which is particularly suitable for large datasets as it reduces the possibility of overfitting. This method is considered an incomplete cross-validation approach. Leave-P-out cross-validation is a more exhaustive cross-validation method. It involves removing *P* samples from the entire dataset to create all possible training and testing sets. For a dataset with *n* samples, this method generates $C_{n}^{p}$ sets of training–testing pairs. In this study, 5-fold cross-validation, 10-fold cross-validation, and leave-P-out cross-validation methods were used for model comparison. The results are shown in Table 7:

The results indicate that the 10-fold cross-validation method performed the best across all evaluation metrics, with the lowest MAE, RMSE, and MAPE values, and the highest *R*^2^ value, reaching 0.9769. This suggests that 10-fold cross-validation not only improves the model’s accuracy but also enhances its generalization ability on unseen data. In comparison, while the 5-fold cross-validation method is relatively simple, it falls slightly short in terms of predictive accuracy. The leave-P-out cross-validation method demonstrated performance similar to the 10-fold method but, due to its computational complexity, may be less efficient for practical applications. Therefore, it is recommended to prioritize 10-fold cross-validation during model evaluation to achieve more reliable predictive results and better model performance assessment.

### 4.4. Ablation Experiments

**Figure 11:**
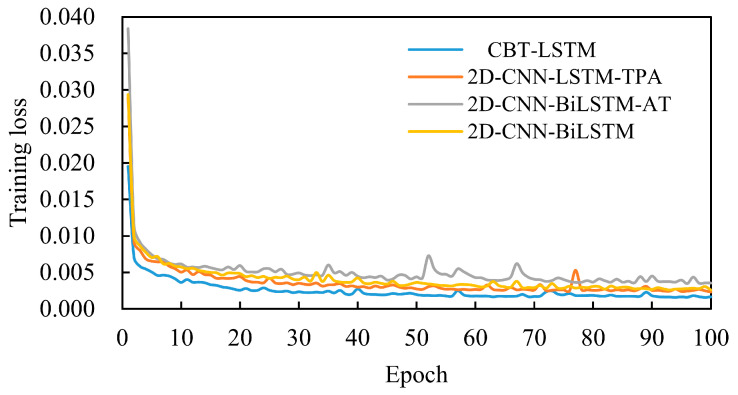
Training loss of the four models.

**Figure 12:**
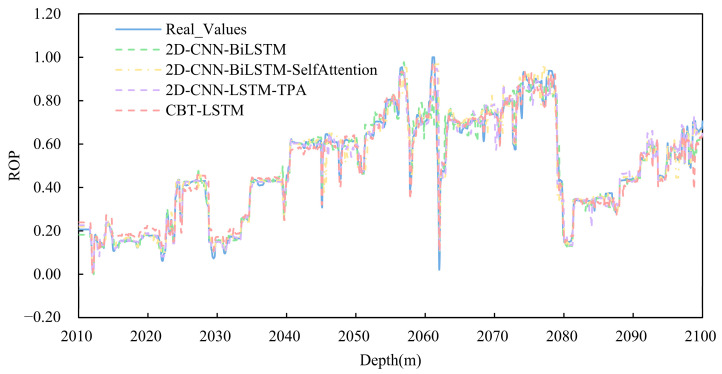
Comparison results of ablation experiments.

To validate the effectiveness of the improvements proposed in this paper, three sets of ablation experiments were designed to evaluate the roles of BiLSTM and TPA. Four models participated in the ablation study: CBT-LSTM, 2D-CNN-LSTM-TPA, 2D-CNN-BiLSTM-SelfAttention, and 2D-CNN-BiLSTM. All other parameters of these models were kept consistent. Figure 11 shows the changes in loss values during the training process for the four models. As seen in the figure, the training loss of the CBT-LSTM model continuously decreases throughout the training process and exhibits lower loss values compared with the other models. The comparison results are shown in Figure 12.

Table 8 shows the comparison of evaluation metrics for ablation experiments on the test set. The figure and table show that the proposed model achieves the highest prediction accuracy. Replacing BiLSTM with a standard LSTM results in decreased prediction accuracy. This is likely because, while LSTM effectively handles time series data, it lacks the bidirectional capability necessary to fully capture complex dependencies. Additionally, the model’s prediction accuracy declines in the absence of an attention mechanism. Replacing TPA with self-attention also leads to a reduction in prediction accuracy. Although the self-attention mechanism is effective, TPA is better at capturing complex temporal variations and key patterns, significantly enhancing the model’s predictive performance. These ablation experiments demonstrate that the inclusion of BiLSTM and TPA plays a crucial role in improving the model’s predictive performance, fully validating the proposed enhancements.

### 4.5. Contrast Experiments

**Figure 13:**
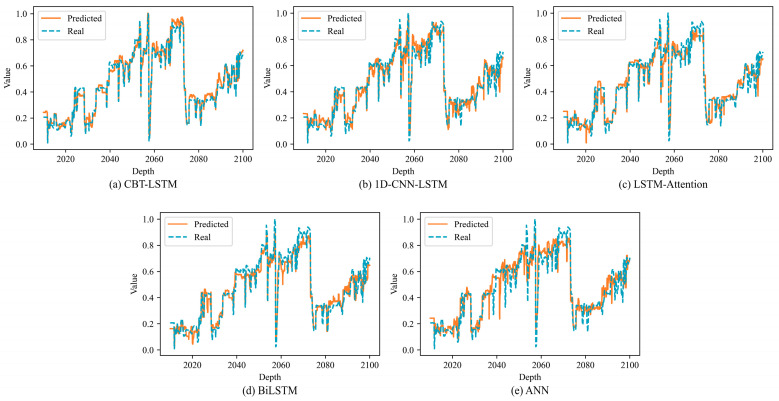
Comparison of the predicted ROPs and the measured ROPs.

To verify the significant advantages of the proposed ROP prediction model over other commonly used ROP prediction models, comparisons were made with 1D-CNN-LSTM, LSTM-Attention, BiLSTM, and ANN algorithms. Figure 13 shows the comparison between the actual ROP values and the predicted ROP values of different models. From the graph, it is evident that the predicted ROP curve obtained by the proposed model aligns more closely with the actual data curve compared with the ROP predictions from other models.

**Figure 14:**
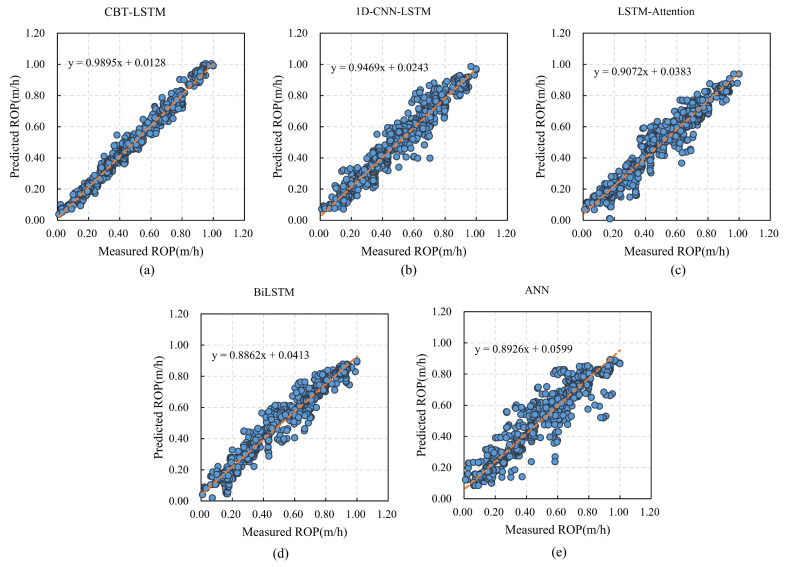
Relationships between the predicted ROPs and the measured ROPs of five models: (**a**) CBT-LSTM; (**b**) 1D-CNN-LSTM; (**c**) LSTM-Attention; (**d**) BiLSTM; (**e**) ANN.

Figure 14 illustrates the linear relationship between the predicted and measured ROP values for five different models. The CBT-LSTM model shows the closest correlation between the predicted and measured ROP, indicating its superior performance. The prediction results of different models are shown in Table 9. After comparing the performance metrics of these five models, the CBT-LSTM model demonstrated the best performance in key evaluation metrics such as $R^{2}$, MAE, MAPE, and RMSE.

The superior performance of the CBT-LSTM model can be attributed in part to its use of a sliding window, which transforms the time-series data into a 2D structure, enabling the 2D-CNN to capture temporal–spatial relationships more effectively than other models. This model achieved the lowest MAE, RMSE, and MAPE, and the highest *R*^2^, indicating its ability to accurately predict ROP with significantly improved accuracy compared with other neural networks. Considering the evaluation of all results, the CBT-LSTM model demonstrates superior performance across all evaluation metrics, further proving its practicality in ROP prediction.

### 4.6. Model Robustness Validation

During the drilling data collection and transmission process, factors such as sensor inaccuracies, unstable communication lines, and electromagnetic interference can lead to measurement errors and data loss, which in turn cause data quality issues. To assess the robustness of the model, noise is typically introduced into the training samples. By comparing the changes in the model’s performance metrics before and after the introduction of noise, the model’s sensitivity to varying levels of noise can be measured. This approach helps evaluate how well the model can handle noisy and incomplete data, which is crucial for ensuring reliable performance in real-world drilling applications.

In the study of the Well A dataset, outliers and missing values in the dataset were already removed and denoised. To further validate the robustness of the model, this research introduced 10%, 20%, and 30% noise and missing values into the original training set. Specifically, the total number of elements to be processed was calculated based on the specified percentages, and random indices were selected for those elements. For each selected index, there was a 50% probability of adding noise and a 50% probability of setting it to a missing value. The noise added was drawn from a normal distribution with a mean of 0 and a standard deviation of 0.1. All hyperparameters were set to their default values. No denoising was applied to the introduced noise. However, as PCA dimensionality reduction cannot handle NaN values, the missing information in the features was filled using polynomial interpolation.

**Figure 15:**
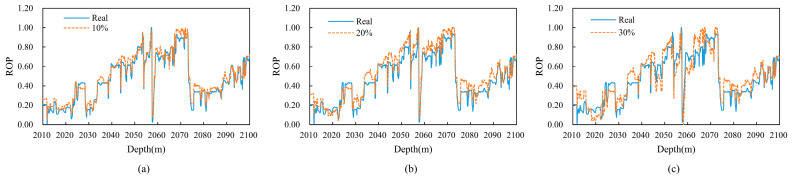
Comparison of predicted values and true values in Well A dataset (after adding different proportions of noise and null values). (**a**) 10% noise and null values added. (**b**) 20% noise and null values added. (**c**) 30% noise and null values added.

Figure 15 shows the comparison between the predicted values and the actual values for the Well A dataset after the addition of noise. Table 10 lists the performance metrics of the model using the original data versus the data with added noise.

From the results presented in the figures and table, it can be observed that adding 10% noise has a minimal impact on the model’s performance. However, as the noise level increases to 30%, the model’s *R*^2^ value decreases significantly, though it remains at 0.8061. This demonstrates that the model retains strong robustness, even with a higher noise level. These findings provide strong support for the model’s application in complex drilling environments, where data quality may be compromised due to various external factors.

### 4.7. Model Generalization Verification

**Figure 16:**
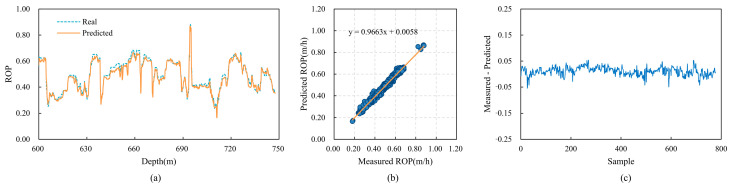
The predictive performance of CBT-LSTM on Well B. (**a**) Comparison between real ROP and predicted ROP. (**b**) Cross-plot of real ROP and predicted ROP. (**c**) Relative error between real ROP and predicted ROP.

**Figure 17:**
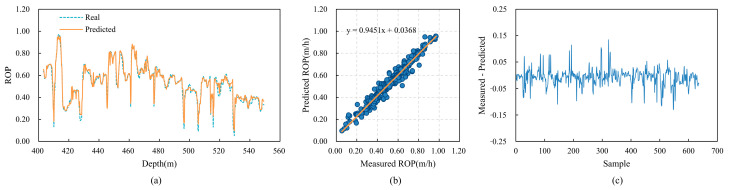
The predictive performance of CBT-LSTM on Well C. (**a**) Comparison between real ROP and predicted ROP. (**b**) Cross-plot of real ROP and predicted ROP. (**c**) Relative error between real ROP and predicted ROP.

**Figure 18:**
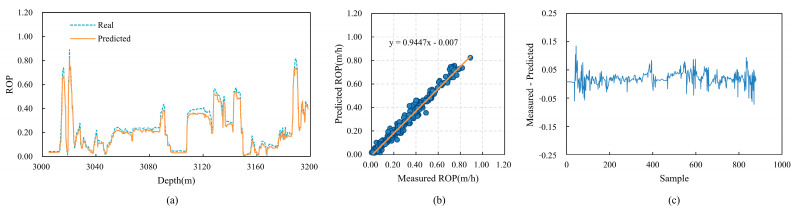
The predictive performance of CBT-LSTM on Well D. (**a**) Comparison between real ROP and predicted ROP. (**b**) Cross-plot of real ROP and predicted ROP. (**c**) Relative error between real ROP and predicted ROP.

To validate the generalization capability of the CBT-LSTM model, it was applied to three other wells (Wells B, C, and D) in the same region for training and testing. The prediction results for these wells are shown in Figure 16, Figure 17 and Figure 18. Figure 16a, Figure 17a and Figure 18a display the fit between the actual ROP and the predicted ROP for the three wells, demonstrating that the two curves generally remain consistent. Figure 16b, Figure 17b and Figure 18b show scatter plots of the actual ROP versus the predicted ROP, with most blue points distributed near the orange line, indicating a strong correlation between the actual and predicted ROP values. Figure 16c, Figure 17c and Figure 18c illustrate the relative error between the actual and predicted values. The errors are relatively small for Well B, and although there are fluctuations for Wells C and D, the errors remain within an acceptable range.

The evaluation metric results are presented in Table 11. The model generally performs well in most cases, especially on the datasets for Wells B and D. The performance is slightly inferior on the Well C dataset, possibly due to the model’s insufficient learning capability for shallower well depths. Overall, the model accurately captures the complex nonlinear relationship between the actual ROP and its features, demonstrating good generalization performance across different wells.

### 4.8. Limitations and Future Directions

Despite achieving high accuracy and generalization capability in ROP prediction through neural networks, the main limitation of this study lies in the scope of the data. First, the various parameters in the drilling process may differ, as different regions and formations involve distinct bit designs, operations, and geological parameters. A mismatch between the model’s training conditions and the actual application environment could lead to a decrease in predictive accuracy. Moreover, the complexity and uncertainty of subsurface geological conditions across different global regions mean that this model has not yet been fully validated in diverse real-world settings, particularly in wells outside the original test area. This constitutes a major limitation of the current research.

In future work, it is necessary to evaluate the model using more generalized datasets, such as data from different oil and gas wells and varied operational scenarios, especially when wells have a high inclination. Additionally, there will be an exploration of integrating cutting-edge deep learning techniques, such as reinforcement learning and transfer learning, to deeply mine the intrinsic patterns in the data and further enhance the model’s predictive accuracy and robustness. Finally, the improved model will be integrated into real-time monitoring systems, enabling real-time safety prediction and monitoring during the drilling process.

## 5. Conclusions

To address the issues of low ROP prediction accuracy and insufficient utilization of data features, this paper proposes a novel ROP prediction model called CBT-LSTM, which integrates 2D-CNN, BiLSTM, and temporal pattern attention (TPA). In this model, 2D-CNN is responsible for extracting complex feature relationships from the processed data, BiLSTM captures bidirectional information within the data, and TPA dynamically assigns feature weights to enhance the network’s ability to extract critical information.

The experiments were conducted using data from four vertical wells in a Chinese oilfield. First, noise was reduced using an SG filter, and features were selected using five different correlation coefficient methods. Principal component analysis (PCA) was then applied for dimensionality reduction, and a sliding window approach was used to convert one-dimensional sequential data into two-dimensional spatial sequence data. By comparing various hyperparameter optimization algorithms and cross-validation methods, the optimal combination was identified. Additionally, ablation experiments were conducted to validate the importance of BiLSTM and TPA in improving the model’s performance.

To validate the effectiveness and potential advantages of the proposed model, a comprehensive comparison was conducted with benchmark models including 1D-CNN-LSTM, LSTM-Attention, BiLSTM, and ANN. The CBT-LSTM model achieved MAE, MAPE, RMSE, and *R*^2^ values of 0.0295, 0.0357, 9.3101%, and 0.9769, respectively, demonstrating higher prediction accuracy than the other models. Additionally, to test the robustness of the model, noise and missing values were introduced into the training data from Well A. When the proportion of outliers was 10%, 20%, and 30%, the model’s *R*^2^ values were 0.9583, 0.8943, and 0.8061, respectively. Although the model’s accuracy declined with increasing noise, it remained above 0.80, indicating strong resilience in handling anomalies, further validating its robustness. Finally, in generalization experiments on the other three wells, the model achieved *R*^2^ values exceeding 0.95, confirming its strong generalization ability across different wells and operational conditions.

By combining theoretical knowledge with practical implementation, this study conducted extensive experiments to validate the effectiveness of the proposed model. The experimental results clearly demonstrate the superiority of the CBT-LSTM model. This research not only provides an effective approach by which to improving ROP prediction accuracy but also offers crucial technical support for the optimization of real-time drilling operations.

## Figures and Tables

**Table 1:** Information on the wells in our datasets.

Well Name	Depth Start (m)	Depth End (m)	Data Entries
Well A	987.2	2280.9	23,896
Well B	490.7	807.6	7802
Well C	301.2	633.3	6389
Well D	2434.5	3338.9	8820

**Table 2:** The details of the parameters of Well A.

Parameters Name	Units	Minimum	Maximum	Average	Standard Deviation
Rate of penetration (ROP)	m·h^−1^	1.531	56.072	29.675	13.589
Measured depth (MD)	m	987.948	2280.910	1643.648	385.767
Weight on bit (WOB)	Kgf	0.785	10.136	4.571	1.601
Average standpipe pressure (ASP)	kPa	6479	20,139	15,715.624	3140.948
Average surface torque (AST)	kN·m	2.113	26.553	10.453	2.888
Average rotary speed (ARS)	rpm	0	180.542	135.550	30.710
Mud flow in (MFI)	L·min^−1^	1581.233	4538.450	4063.844	412.429
Mud density in (MDI)	g·cm^−3^	1.022	1.362	1.252	0.072
Average hookload (AH)	Kgf	100.666	148.950	125.547	15.409
Hole depth (TVD)	m	987.464	2279.373	1643.106	385.687
Gamma (G)	GAPI	12.941	226.280	85.365	28.976

**Table 3:** Correlation coefficients between the ROP and the features.

Features	Sc	Kc	Dc	Mc
MD	0.053	0.035	0.257	0.893
WOB	−0.110	−0.094	0.320	0.483
ASP	0.146	0.096	0.302	0.417
AH	0.285	0.184	0.326	0.492
TVD	0.053	0.035	0.257	0.893
G	0.150	0.098	0.187	0.303

**Table 4:** The impact of different convolution kernel combinations on 2D-CNN model performance.

Kernel	Number Kernels	MAE (m/h)	RMSE (m/h)	MAPE (%)	$R^{2}$
2 × 1	{32, 32, 32}	0.0442	0.0580	11.8018	0.9542
	{64, 64, 64}	0.0325	0.0488	10.8124	0.9624
	{128, 128, 128}	0.0497	0.0672	12.7291	0.9428
3 × 1	{32, 32, 32}	0.0385	0.0530	11.3076	0.9573
	{64, 64, 64}	0.0353	0.0518	11.0714	0.9596
	{128, 128, 128}	0.0461	0.0595	12.3825	0.9499
3 × 3	{32, 32, 32}	0.0396	0.0545	11.5324	0.9563
	{64, 64, 64}	0.0417	0.0573	11.7311	0.9555
	{128, 128, 128}	0.0469	0.0625	12.4218	0.9446

**Table 5:** Search scope of hyperparameter.

Hyperparameter	Value
Batch size	[$2^{4},2^{5},2^{6},2^{7},2^{8}$]
Learning rate	[$10^{-1},\;10^{-2},{\;5\;\times \;10}^{-3},\;{1\;\times \;10}^{-3},\;{5\;\times \;10}^{-4}$]
Dropout1	[0.1, 0.2, 0.3, 0.4, 0.5]
Dropout2	[0.1, 0.2, 0.3, 0.4, 0.5]

**Table 6:** Best hyperparameters identified by different search methods.

Search Methods	*R* ^2^	MAE (m/h)	Batch Size	Learning Rate	Dropout1	Dropout2
Grid search	0.9630	0.0327	64	0.001	0.2	0.4
Random search	0.9652	0.0316	64	0.005	0.3	0.2
PSO	0.9684	0.0301	64	0.005	0.2	0.2

**Table 7:** Performance comparison of different cross-validation methods.

Cross-Validation Methods	MAE (m/h)	RMSE (m/h)	MAPE (%)	*R* ^2^
5-fold	0.0325	0.0392	9.7690	0.9712
10-fold	0.0295	0.0357	9.3101	0.9769
Leave-P-out	0.0316	0.0368	9.4710	0.9756

**Table 8:** Error comparison of prediction results in ablation experiments.

Model	MAE (m/h)	RMSE (m/h)	MAPE (%)	*R* ^2^
CBT-LSTM	0.0295	0.0357	9.3101	0.9769
2D-CNN-LSTM-TPA	0.0350	0.0442	10.5011	0.9649
2D-CNN-BiLSTM-SelfAttention	0.0368	0.0532	10.8816	0.9596
2D-CNN-BiLSTM	0.0364	0.0579	11.0015	0.9589

**Table 9:** Comparison of performance evaluation metrics of five models.

Model	MAE (m/h)	RMSE (m/h)	MAPE (%)	*R* ^2^
CBT-LSTM	0.0295	0.0357	9.3101	0.9769
1D-CNN-LSTM	0.0415	0.0542	13.0366	0.9468
LSTM-Attention	0.0459	0.0601	14.6316	0.9346
BiLSTM	0.0453	0.0569	13.7240	0.9413
ANN	0.0552	0.0782	18.1144	0.8892

**Table 10:** Comparison of performance indicators between the original data and the model with added noise.

Model	MAE (m/h)	RMSE (m/h)	MAPE (%)	*R* ^2^
CBT-LSTM	0.0295	0.0357	9.3101	0.9769
10%	0.0404	0.0479	13.9782	0.9583
20%	0.0665	0.0764	22.2350	0.8943
30%	0.0899	0.1035	31.6087	0.8061

**Table 11:** Evaluation results of the model on three wells.

Well Name	MAE (m/h)	RMSE (m/h)	MAPE (%)	*R* ^2^
Well B	0.0089	0.0130	2.1395	0.9865
Well C	0.0214	0.0581	3.4097	0.9534
Well D	0.0175	0.0230	8.6426	0.9813

## Data Availability

The dataset used in this study is not publicly available. Researchers with requests should contact the corresponding author. We will provide support and additional information within reasonable boundaries.
